# The *Pseudomonas aeruginosa* lectin LecB binds to the exopolysaccharide Psl and stabilizes the biofilm matrix

**DOI:** 10.1038/s41467-019-10201-4

**Published:** 2019-05-16

**Authors:** Daniel Passos da Silva, Michael L. Matwichuk, Delaney O. Townsend, Courtney Reichhardt, Doriano Lamba, Daniel J. Wozniak, Matthew R. Parsek

**Affiliations:** 1 Department of Microbiology, University of Washington, Seattle, WA USA; 2 Istituto di Cristallografia, Consiglio Nazionale delle Ricerche, Sede Secondaria di Basovizza, Trieste, Italy; 3 Departments of Microbial Infection and Immunity, Microbiology, Ohio State University, Columbus, OH USA; 4 Integrative Microbiology Research Centre, South China Agricultural University, 510642 Guangzhou, China

**Keywords:** Biofilms, Pathogens

## Abstract

*Pseudomonas aeruginosa* biofilms are composed of exopolysaccharides (EPS), exogenous DNA, and proteins that hold these communities together. *P. aeruginosa* produces lectins LecA and LecB, which possess affinities towards sugars found in matrix EPS and mediate adherence of *P. aeruginosa* to target host cells. Here, we demonstrate that LecB binds to Psl, a key matrix EPS, and this leads to increased retention of both cells and EPS in a growing biofilm. This interaction is predicted to occur between the lectin and the branched side chains present on Psl. Finally, we show that LecB coordinates Psl localization in the biofilm. This constitutes a unique function for LecB and identifies it as a matrix protein that contributes to biofilm structure through EPS interactions.

## Abstract (web summary)

Pseudomonas aeruginosa biofilms are composed of exopolysaccharides, exogenous DNA, and proteins. Here, da Silva et al show that protein LecB binds to matrix exopolysaccharide Psl, thus contributing to increased retention of bacterial cells and exopolysaccharide in growing biofilms.

## Introduction

Biofilms are multicellular aggregates of microbes that are enclosed in a matrix composed of exopolysaccharides (EPS), exogenous DNA (eDNA), and proteins. The extracellular matrix is thought to hold these communities together as well as contribute to bacterial persistence at infection sites by protecting against the host immune system and antimicrobial stresses^1,2^.

The biofilm matrix produced by non-mucoid *Pseudomonas aeruginosa* strains primarily contains two EPS, Pel, and Psl, which form a scaffold that maintains biofilm structure^3–5^. Pel recently was described as an *N*-acetyl glucosamine (GlcNAc)- and *N*-acetyl galactosamine (GalNAc)-rich polysaccharide that is charged under slightly acidic pH and interacts with eDNA in the matrix^6^. Psl is composed of a neutral pentasaccharide subunit that contains mannose, rhamnose, and glucose in a 3:1:1 ratio^4,7^. The levels of these polysaccharides within the matrix and their relevance for aggregate structural stability varies across *P. aeruginosa* strains^5^. Furthermore, these EPS can be found as both cell-associated and secreted forms.

Less is known concerning the identity and function of *P. aeruginosa* biofilm matrix proteins. Proteins that interact with these EPS can contribute to biofilm structural integrity and maintenance. To date, the best described matrix protein is the extracellular adhesin CdrA, which promotes aggregate formation through Psl interactions under planktonic conditions, and helps to stabilize the matrix and maintain aggregate structural integrity^3^. It was recently shown that CdrA can also promote bacterial aggregation in the absence of EPS^8^. Outside of CdrA, no other matrix proteins that play a role in biofilm structural stability have been identified.

*P. aeruginosa* produces two small soluble lectins, LecA and LecB (also named PAI-L and PAII-L, respectively) that interact with specific sugars. Crystal structures have been solved for both, and binding affinity experiments showed that LecA binds to galactose and its derivatives, while LecB binds to fucose, mannose, and mannose-containing oligosaccharides^9–12^. In addition, it is important to note that functional LecB is a homotetramer consisting of four 114 amino acid LecB monomers which require two divalent calcium ions and has been shown to be associated with the outer membrane^9,10,13^. The primary functional roles attributed to these lectins is to mediate attachment to the host during infection. In particular, it was shown that LecA is involved in host cell invasion and cytotoxicity, while LecB reduces ciliary beating of airway epithelium^14–16^. Both lectins also are linked to biofilm formation on abiotic surfaces, although the underlying mechanism behind these observations are unknown^13,17^. Culturing *P. aeruginosa* in the presence of the monosaccharides that are the binding partners for these lectins inhibits biofilm maturation^13,16,18,19^. This discovery led to the development of putative therapeutic approaches using glycomimetics that disrupt LecB-sugar interactions^15,20–23^.

Interestingly, Psl contains mannose, a target monosaccharide for LecB. In this study, we demonstrate that LecB binds to Psl. We then show that LecB positions Psl within the matrix and that this interaction is crucial for aggregate formation. We find that, unlike biotic surfaces, LecB is not important for adhesion to abiotic surfaces, but its presence leads to increased retention of cells and EPS in the biofilm. This study identifies LecB as a *P. aeruginosa* biofilm matrix protein that binds to Psl and promotes cell retention.

## Results

### LecB binds to the Psl exopolysaccharide

Pel and Psl are biofilm matrix EPS that are crucial for *P. aeruginosa* biofilm formation and structural integrity^5^. LecB binds the monosaccharides fucose and mannose^10,11^. Since mannose residues are present in the pentasaccharide subunit of Psl, we hypothesized that LecB binds to Psl and that this might influence *P. aeruginosa* biofilm structure.

**Fig. 1:**
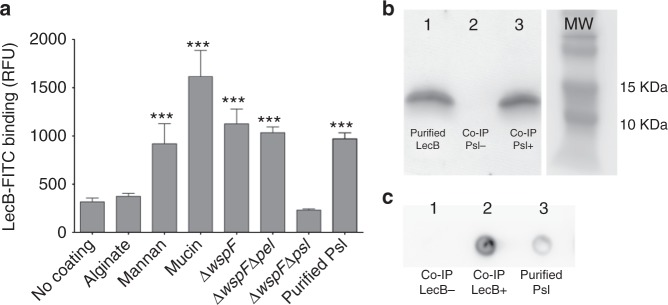
LecB binds to the exopolysaccharide Psl. **a** FLLA assay shows that  LecB is able to bind to fucose/mannose-containing polysaccharides immobilized on the wells of microtiter plates. FITC-conjugated LecB does not bind to uncoated or glucose-rich polysaccharide alginate, and Pel-rich materials (Δ*wspF* Δ*psl*). However, it binds to mannan, fucose-rich proteins (mucin), Psl-rich materials (Δ*wspF* and Δ*wspF* Δ*pel*), and to purified Psl. ***p < 0.0001, *t*-test, *n* > 3. **b** LecB western blot showing coimmunopreciptation of LecB and Psl. Lane 1 was loaded with purified LecB. PtnG Dynabeads coated with anti-Psl antibodies were incubated with LecB (lane 2) or purified Psl and LecB (lane 3). Eluted material was immunoblotted using anti-LecB antibodies. **c** Psl blot showing coimmunopreciptation of LecB and Psl. Protein G Dynabeads coated with anti-LecB antibodies were incubated with purified LecB (lane 1) or with LecB and Psl (lane 2). After wash steps, eluted material was immunoblotted using anti-Psl antibodies. Lane 3 was loaded with purified Psl

To test this hypothesis, we quantified the binding of purified LecB to Pel and Psl. For this binding assay, we used purified Psl as well as crude polysaccharide preparations from the following strains: Δ*wspF* (overproduces Pel and Psl), Δ*wspF* Δ*pel* (overproduces Psl only), and Δ*wspF* Δ*psl* (overproduces Pel only). Fluorescently labeled LecB (LecB-FITC) was used in a fluorophore-linked lectin assay (FLLA), in which LecB-FITC was tested for binding to microtiter plate wells that were coated with exopolysaccharide preparations. We observed that LecB bound to wells coated with purified Psl as well as those coated with EPS preparations from Δ*wspF* and Δ*wspF* Δ*pel* but not Δ*wspF* Δ*psl* (Fig. 1a). As expected, LecB-FITC also bound to wells coated with the positive controls mannan (a mannose-rich polysaccharide) and mucin (highly glycosylated proteins, decorated with fucose residues). LecB-FITC did not interact with wells coated with the negative control alginate, which does not contain either mannose or fucose.

As a complementary approach to investigate LecB–Psl interactions, we performed co-immunopreciptations (Co-IP) using beads coated with either anti-Psl or anti-LecB antibodies. When anti-Psl coated beads were incubated with purified Psl and purified LecB, we detected LecB in the eluted fraction only when both Psl and LecB were present in the input fraction (Fig. 1b). Previously, we had shown that the type VI secretion effector Tse1 is an extracellular protein that does not bind Psl^24^, and thus serves as a negative control. We also performed Co-IPs using beads conjugated to anti-LecB antibodies which were subsequently incubated with purified LecB and Psl. An anti-Psl immunoblot was used to evaluate for the presence of Psl in the eluted fraction. A positive Psl signal was observed only when both Psl and LecB were present in the input fraction (Fig. 1c). As expected the Tse1 negative control did not bind Psl (data not shown). Together, these results support that LecB binds to Psl.

### LecB binds to a branched mannose residue on the side chain of Psl

**Fig. 2:**
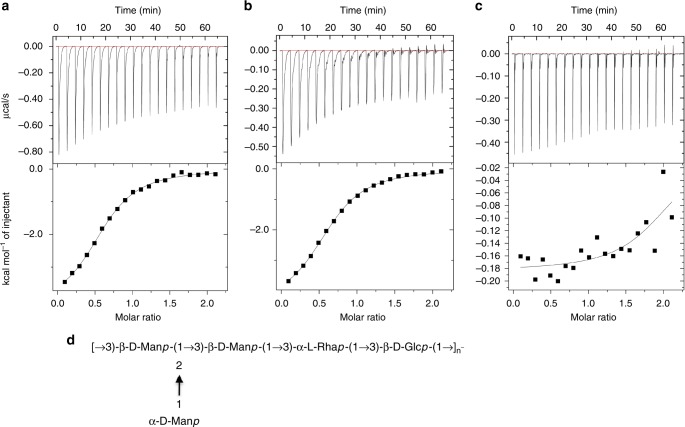
LecB binds α-mannobiose, but not β-mannobiose. Titration of **a** α-1,2′ mannobiose (Psl side chain) into purified LecB, **b** α-1,3′ mannobiose into purified LecB (not present in Psl), and **c** β-1,3′ mannobiose (Psl linear chain) into purified LecB. **d** Chemical structure of the Psl displaying the α-1,2′ mannobiose side chain and β-1,3′ mannobiose in linear chain

We sought to examine the specific nature of LecB–Psl interactions. Toward this end, we quantified the binding of purified LecB to relevant disaccharides by isothermal titration calorimetry (ITC). We hypothesized that LecB would interact with one or more of the mannose residues found in Psl. There are three linked mannose residues present in the repeating pentasaccharide unit of Psl. Two mannose residues are in the linear polysaccharide chain and are linked via β-1,3′ glycosidic bonds. The third mannose residue branches from the linear chain and is connected by α-1,2′ linkage to a mannose residue in the linear chain (Fig. 2d).

For ITC experiments we purified LecB overexpressed in *P. aeruginosa* using affinity chromatography as previously described^13^. For each ligand, the binding constants (*K*_d_) and thermodynamics were determined from three independent experiments (Table 1, Supplementary Fig. 8). LecB bound to α-1,2′ mannobiose with a *K*_d_ of approximately 27 µM (Fig. 2a), which is comparable to the values reported in the literature for LecB-mannose interactions^11^. However, LecB did not bind to β-1,3′ mannobiose (Fig. 2c). The α-1,2′ mannobiose disaccharide is present in the Psl side chain, while β-1,3′ mannobiose is part of the linear polysaccharide chain. Interestingly, we found that LecB binds to α-1,3′ mannobiose with a similar binding affinity (25 µM) as to α-1,2′ mannobiose (Fig. 2b). This indicates that the β-linkage and not the C3 linkage is impeding interactions between LecB and the non-reducing mannose. As expected, the negative control maltose (α-1,4′ glucobiose) did not interact with LecB (Table 1).

**Table 1:** ITC analysis for binding of LecB to different disaccharide

Disaccharide	*K*_d_ (µM)	−Δ*G* (kJ/mol)	−Δ*H* (kJ/mol)	−*T*Δ*S* (kJ/mol)	*n*
α-1,2′ mannobiose	26.60 ± 2.61	26.09 ± 0.21	20.71 ± 2.59	5.38 ± 2.75	0.62 ± 0.01
α-1,3′ mannobiose	33.63 ± 7.18	25.58 ± 0.57	20.54 ± 1.57	5.05 ± 2.12	0.61 ± 0.03
β-1,3′ mannobiose	n.d.	n.d.	n.d.	n.d.	n.d.
Methyl-α-D-mannoside^11^	71	23.7	17.8	5.9	0.94
Maltose	n.d.	n.d.	n.d.	n.d.	n.d.

Values are mean ± S.D. from three separate titrations. n.d. indicates that with ligand concentrations used were not enough to determine these values

**Fig. 3:**
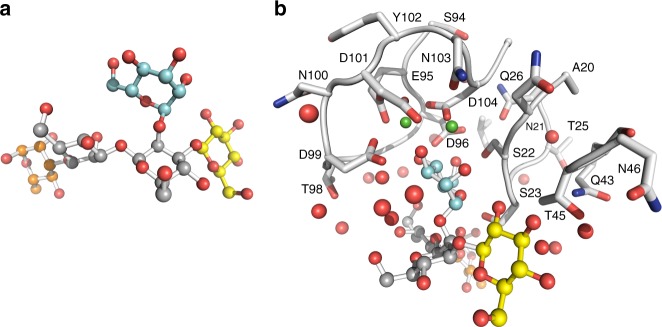
Modeling of LecB–Psl interactions favors binding to the side chain of Psl. **a** Overall conformation of the docked Psl pentasaccharide is represented as α-D-mannopyranose in cyan, β-D-mannopyranose in gray, α-L-rhamnopyranose in orange, and β-D-glucopyranose in yellow. Oxygen atoms are labeled in red. **b** Structural model of Psl docking in LecB binding site is favored to occur in the α-1,2′ mannobiose. LecB is depicted in white, water molecules are the red spheres, the green ones are Ca^+2^ ions, and Psl follows the same color code from (**a**)

These results led us to hypothesize that LecB would preferentially bind to the mannose present on the side chain of Psl. To explore this idea, we performed molecular docking simulations using as protein receptor template the crystal structure of LecB in complex with D-mannose^25^ and the theoretical model, based on NMR data, of the pentasaccharidic repeating unit of Psl^4,26^. Minimal manual rebuilding of the model of LecB–Psl complex were needed in order to properly dock the Psl (Fig. 3a) side chain (α-1,2′ mannobiose) to the binding site of LecB (Fig. 3b). In contrast, major structural changes were required to prevent major steric clashes between LecB and Psl when the linear chain segment (β-1,3′ mannobiose) was tentatively docked into LecB binding site.

 A major feature of the Psl-binding site of LecB is a pair of calcium ions. The α-D-Man*p* sugar side chain, adopts the lowest energy ^4^C_1_ conformation, and via its hydroxyl groups interacts with either (O2 2.7 Å), (O4 2.8 Å) or both (O3 2.5 Å; 2.6 Å) the calcium ions (Supplementary Fig. 1). The protein residues involved in the coordination of the two calcium ions, 3.7 Å apart from each other, belong to a single PAO1 LecB loop encompassing residues Asn95-Asp104 and comprises also residue Asn21 (Fig. 3a). The bound Psl α-D-Man*p* sugar side chain interacts via hydrogen bonds with PAO1 LecB Ser22 (2.2 Å) and Ser23 (3.1 Å, 3.3 Å) (Suplementary Fig. 1).

The role of bridging water and hydrogen bonding as key determinants of non-covalent protein-carbohydrate recognition has been recently reviewed^27^. Indeed, two water molecules are engaged at the PAO1 LecB–Psl interface (Supplementary Fig. 1). The first, besides interacting with the second water molecule (2.9 Å) and the O4 hydroxyl of the α-D-Man*p* sugar side chain (2.7 Å) also interacts with the PAO1 LecB residues Thr98 (2.9 Å) and Asp99 (2.4 Å; 3.1 Å). The second water molecule does not get involved in protein contacts, but interacts with the O5 oxygen (2.9 Å) of the pyranose ring and with the methyl hydroxyl O6 (3.0 Å) of the β-D-Man*p* sugar side chain.

It is worthy to note that the predicted PAO1 LecB Psl docking nicely superimpose (Supplementary Fig. 2) on the previously reported crystal structure of the PA14 LecB in complex with the D-Man*p*-α-1,3-D-Man*p* disaccharide (PDBID: 5A6Y)^23^. The protein residues involved at the Psl-binding site are highly conserved and only differ at positions 23 (PA14 LecB Ser; PAO1 LecB Ala) and 97 (PA14 LecB Ser; PAO1 LecB Gly) with an overall r.m.s.d. (23 C^α^ atoms) of 0.11 Å. The binding orientation of D-Man*p*-α-1,2-D-Man*p* fragment of the Psl pentasaccharide in the computed complex with PAO1 LecB closely resembles that of the D-Man*p*-α-1,3-D-Man*p* disaccharide in complex with PA14 LecB, validating the previously reported IC_50_ binding affinities of either disaccharides^23^ as well as the reported ITC thermodynamic signatures in our present work. Collectively, these results indicate that binding to the linear chain of Psl is unlikely, and Psl structure heavily favors binding of PAO1 LecB to its side chain.

### LecB binds in situ to Psl in the biofilm matrix

Upon discovering that LecB binds Psl, we hypothesized that the lectin could also bind to Psl found in the context of the biofilm matrix. Since Psl is already known to interact with other matrix components, we were unsure if LecB binding sites would be accessible. To test our hypothesis, we applied fluorescently labeled LecB-FITC along with HHA-TRITC, a plant lectin known to specifically stain Psl^28,29^ to 4-day-old biofilms and monitored the relative staining patterns for the wild-type strain PAO1, and the negative control strains PAO1 Δ*psl* and PA14 (another laboratory wild-type strain that is incapable of producing Psl and to compensate utilizes Pel)^5^.

**Fig. 4:**
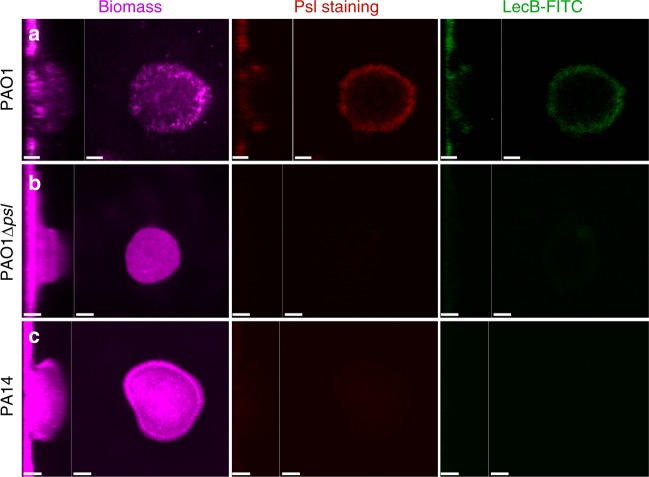
LecB binds mature biofilms in Psl-rich regions. Exogenously added FITC-conjugated LecB binds to biofilms containing Psl. **a** PAO1 4-day-old biofilm stained with Syto 62 (magenta), HHA-TRITC (red), and LecB-FITC (green) showed that LecB binds to similar regions as HHA. **b** Although PAO1 Δ*psl* produces very few aggregates relative to PAO1, those that were found were negative for HHA and LecB staining. **c** The same was observed for a PA14 biofilm, which cannot produce Psl, but still makes Pel. Scale bars = 25 µm

As shown in Fig. 4a, we observed the characteristic Psl staining pattern present at the periphery of biofilm aggregates of PAO1. LecB-FITC staining was observed in the same Psl-rich regions. As expected in strains PAO1 Δ*psl* and PA14 (Fig. 4b, c, respectively), which are unable to produce Psl, we did not observe staining with either HHA-TRITC or LecB-FITC. These results suggest that LecB is able to interact with Psl that is present in the biofilm matrix environment.

### LecB coordinates Psl localization within the matrix

**Fig. 5:**
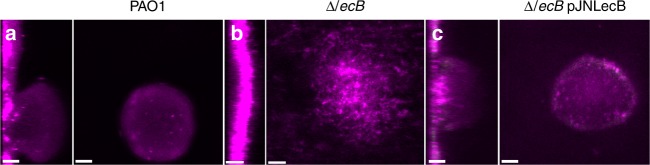
LecB mutants display a defect on mature aggregate formation under flow conditions. **a** PAO1 4-day-old biofilm displays a complex mature structure in NB media while **b** Δ*lecB* 4-day-old biofilm do not fully develop into mature structures. **c** Complemented strain Δ*lecB* pJNLecB 0.05% arabinose 4-day-old biofilm develop structures that are very similar to PAO1. Biofilms are stained for biomass with Syto 62 at 2.5 µM. Scale bars = 25 µm

We next sought to determine the impact of a *lecB* mutation on biofilm structure under constant flow conditions. When biofilms were cultured in a dilute complex growth medium (NB 1.4% v/v), the wild type formed biofilms characterized by numerous cellular aggregates that extended > 50 microns above the attachment surface (Fig. 5a), while the Δ*lecB* strain produced a monolayer of cells punctuated infrequently by small mounds of cells that did not usually extend more than 25 microns above the surface (Fig. 5b). To quantify this phenotypic difference, we applied the image analysis software COMSTAT. Surface roughness is a key measurement made by COMSTAT that illustrates the numerous aggregates present in the wild type, but are absent from the mutant. COMSTAT analysis revealed that wild-type biofilms were considerably rougher than the *lecB* mutant (Supplementary Fig. 3). Complementation of the Δ*lecB* mutation in trans largely restored the wild-type biofilm phenotype (Fig. 5c and Supplementary Fig. 3). These results suggest that LecB plays a role in producing and maintaining biofilm aggregates.

**Fig. 6:**
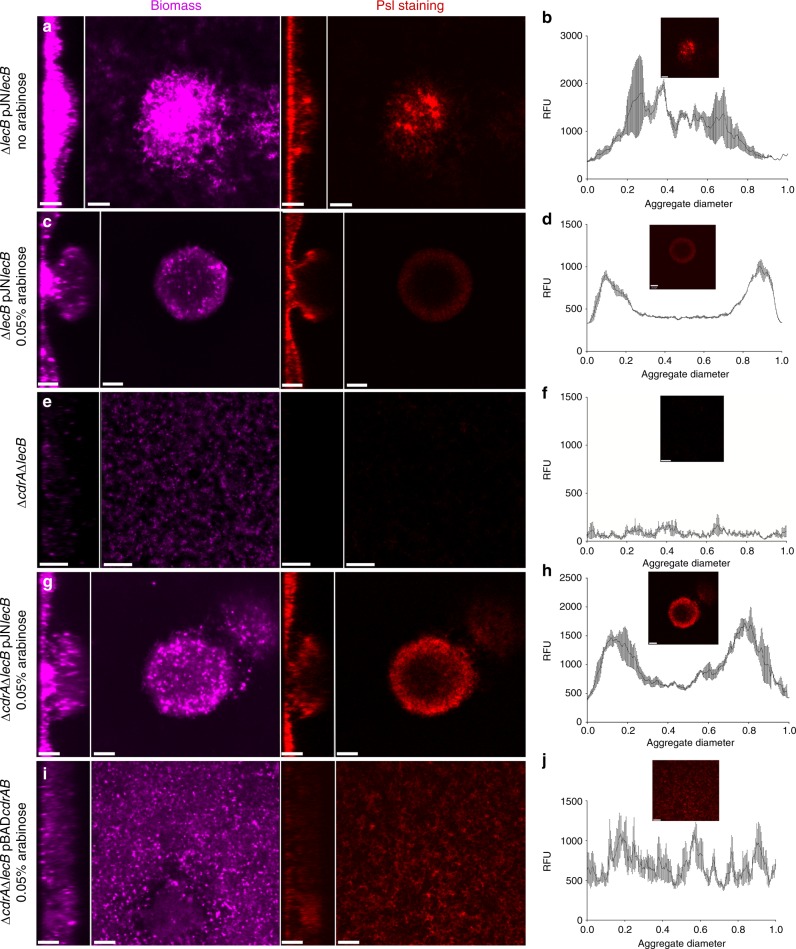
LecB coordinates Psl positioning in the matrix. **a** Syto 62 (magenta) and HHA-TRITC (red) stained biofilms of Δ*lecB* pJNLecB grown in NB without arabinose forms a flat biofilm with **b** Psl distribution being fairly homogenous throughout the biomass (the *x*-axis, RFU, corresponds to the intensity of PSL staining.). Psl intensity profiles were generated from an average of nine independent micrographs. Inset is a representative Psl staining micrograph that was used to generate the profile. **c** Biofilms of Δ*lecB* pJNLecB grown in NB with the addition of 0.05% arabinose present a similar structure and **d** Psl distribution to the ones formed by PAO1. **e** Syto 62 (magenta) and HHA-TRITC (red) stained biofilms of Δ*cdrA* Δ*lecB* pJNLecB grown in NB without arabinose display a loose carpet of cells with **f** no particular Psl localization pattern. **g** Δ*cdrA* Δ*lecB* pJNLecB grown in NB with the addition of 0.05% arabinose rescues PAO1 phenotype and **h** usual Psl placement. **i** Biofilms of Δ*cdrA* Δ*lecB* pBADCdrAB grown in NB with the addition of 0.05% arabinose results in the formation of an undifferentiated thick layer of cells with **j** no particular Psl organization. Psl distribution measurements were performed in nine images from three different experiments per condition. Scale bars = 25 µm

Following our discovery that LecB binds Psl, we hypothesized that the *lecB* mutant strain might exhibit mislocalization of Psl in the matrix. Past work with the Psl-binding matrix protein CdrA showed that aberrant Psl localization in the matrix can accompany observed biofilm structural defects^3^. To address this possibility, we monitored Psl localization patterns in biofilms formed by the LecB conditional expression strain, Δ*lecB* pJNLecB. In the absence of *lecB* expression, we observed that biofilms failed to produce large aggregates and also had no distinct pattern in Psl localization (Fig. 6a). Semi-quantification of Psl levels across the biofilm aggregate indicated that its distribution was random and uneven (Fig. 6b). The same strain, complemented by expressing *lecB*, produced large cellular aggregates that exhibited peripheral Psl localization (Fig. 6c, d), similar to what is seen in the aggregates of wild-type biofilms, although the amount of Psl made in both the presence and absence of arabinose appeared to be comparable.

Previous studies have shown that another matrix protein, CdrA, influences biofilm aggregate formation through Psl interactions^3^. This raises the question as to whether LecB and CdrA are functionally interchangeable? Therefore, we examined if CdrA influenced Psl distribution and aggregate formation in our system. First, we characterized a PAO1 Δ*cdrA* Δ*lecB* double mutant strain, and observed that it produced biofilms composed of a carpet of cells, which contained very little Psl (Fig. 6e, f). In the rare instances where aggregates were observed, they were more susceptible to shear stress in comparison to PAO1 wild type or Δ*lecB* (Movies S1, S2, and S3). Expression of *lecB* in trans in the double mutant background was sufficient to restore wild-type aggregate formation and Psl distribution (Fig. 6g, h). In contrast, expression of *cdrA* in the same background was not sufficient to restore wild-type biofilm characteristics. This strain produced a flat, thick mass devoid of aggregates (Fig. 6i), although it still retained Psl (Fig. 6j). Taken together, these results suggest that when grown in NB, CdrA can influence biofilm structure and Psl retention, but LecB is the primary matrix protein responsible for Psl localization and aggregate production.

Our previous work demonstrated a prominent role for CdrA in aggregate formation. In attempting to reconcile our current results with this past study, we noted that the main culturing difference involved the growth medium. In this study, a dilute complex medium (NB medium) was used to mimic the previous work done on LecB^13^. On the other hand, our past work with CdrA involved dilute LB medium. Indeed, when we compared biofilm phenotypes of PAO1 and the Δ*lecB* mutant strain grown on dilute LB, we found that the mutant strain was able to produce aggregates like the wild type (Supplementary Fig. 4). Since our results suggest that LecB and CdrA can carry out similar roles in the matrix, we hypothesized that either CdrA or LecB expression levels may change significantly when *P. aeruginosa* is grown on NB medium as opposed to LB. The idea being that LecB may not be required under conditions where CdrA is produced at a sufficiently high level (or vice versa). To test this possibility we probed biofilm biomass harvested from the surface of silicone tubing using both CdrA and LecB antisera. We found that biofilm growth on NB produced significantly less CdrA in the matrix than growth on LB. LecB levels were observed to be roughly the same for the two growth media (Supplementary Fig. 5). These data indicate that LecB production is critical for aggregate formation under culturing conditions (e.g., growth on NB) that result in low levels of CdrA expression. The underlying cause of lower CdrA expression levels during growth on NB remains unclear and is a focus of future study.

### LecB promotes retention of Psl and cells within biofilms

**Fig. 7:**
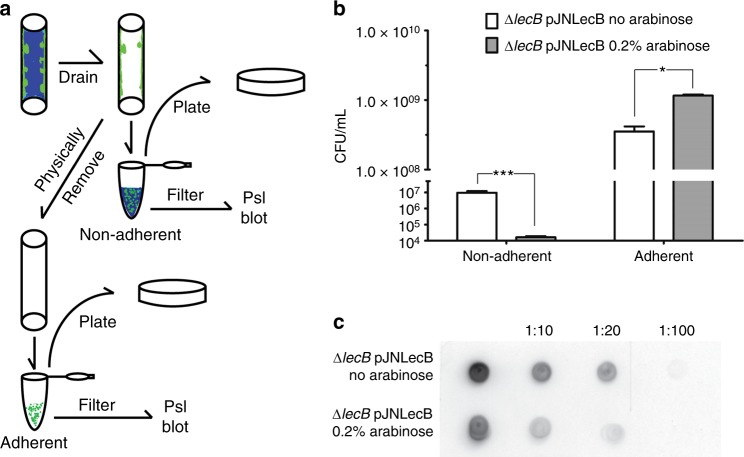
LecB retains cells and matrix material in adherent biomass. **a** Schematics of procedures performed in the samples from the tube biofilm experiment. Tubes are represented containing biomass (green) and media (blue). **b** Quantification of non-adherent and adherent Δ*lecB* pJNLecB cells without or with 0.2% arabinose. Absence of LecB results in increased number of non-adherent cells compared with when LecB is present. Expression of *lecB* increases the number of cells adhered to the tube surface compared with when LecB is not present. ***p < 0.0001, *p *<* 0.005 *t*-test, *n* > 3. **c** Dot blot of non-adherent fractions derived from Δ*lecB* pJNLecB without or with 0.2% arabinose 6-day-old tube biofilms. Immunodetection was performed using anti-Psl antibodies. Expression of *lecB* leads to decreased levels of released Psl when compared with the uninduced condition

Our results indicate that LecB binds Psl and guides its localization within biofilm aggregates. Past work with CdrA demonstrated that its expression was linked to retention of cells and Psl to a growing biofilm. This led us to hypothesize that LecB might play a similar role during the course of biofilm growth. To test this hypothesis, we used the arabinose-inducible *lecB* expression strain and quantified the amount of cells and Psl released into the bulk liquid in the presence and absence of arabinose. To facilitate quantitation of biofilm biomass, the biofilms were cultured in silicone tubes through which media was continuously flown. Initially, we verified by microscopy that the structure of the biofilm formed by the wild-type and *lecB* mutant strains were similar to their flow-cell counterparts (Supplementary Fig. 6). We then determined cell numbers (CFU) and Psl levels for two fractions that were collected from the tubes: (i) loosely attached cells, or “non-adherent” cells, and (ii) firmly attached cells, or “adherent” cells (Fig. 7a).

Our analysis indicates that LecB expression had repercussions that extended beyond the ability to form aggregates. We found that LecB had a profound impact on the ability to retain growing biomass and secreted Psl (Fig. 7b). When LecB was expressed, the number cells was higher in the adherent fraction compared with when it was not expressed (Fig. 7b). When assaying for Psl, we observed lower levels in the non-adherent fractions of the arabinose-induced strain, indicating that LecB also influences the retention of Psl (Fig. 7c), although Psl levels in the adherent fraction were similar whether LecB was present or not (Supplementary Fig. 7). Taken together, these results show that LecB enhances the retention of both cells and Psl to the growing biofilm.

## Discussion

Lectins are found in all domains of life, and their key function is often to mediate interactions^30,31^. In bacteria, lectins are nearly exclusively studied in the context of bacterium interactions with higher Eukaryotes^16,18,32–34^. Indeed, these interactions have critical roles in both disease and symbioses. However, particularly in bacterial species, lectins functioning in the context of microbial communities has not been extensively explored. This is certainly the case for *P. aeruginosa*, where the roles of its two self-produced lectins are largely attributed to disease-related host interactions^16,18^. Our findings suggest that lectin-mediated interactions that stabilize the biofilm matrix represent a distinct function for at least one of these lectins.

Biofilm formation usually involves the production of EPS and proteins that lend structural integrity to the matrix^5,35–38^. One of the better characterized systems demonstrating these principles involves *Vibrio cholerae*. Three matrix proteins were identified in *V. cholerae* that contribute to biofilm stability. After the production of the main exopolysaccharide VPS^39^, a protein involved in cell–cell and cell–surface adhesion (RmbA) accumulates at the cell surface^40–42^. Next, Bap1 is secreted and is thought to crosslink unknown matrix components and cells to ensure matrix integrity, as well as to contribute to the hydrophobicity of the pellicle^41–43^. Last, RbmC accumulates at discrete sites and is crucial for retaining VPS throughout the biofilm^41,42,44^. These principles appear to be conserved in *P. aeruginosa*, with Pel and Psl performing the role of VPS and CdrA and LecB emulating RbmA, Bap1, and RbmC functions.

Biofilm aggregates likely serve some key functions. For biofilms growing at a liquid–solid interface, aggregates protrude out of the boundary layer found at the surface and into the flow stream overlying bulk liquid. One consequence of this is that cells positioned toward the top of the aggregate have favorable access to the overlying nutrients. Indeed, this point is described in a number of laboratory and computational studies of biofilms^45–47^. Aggregates also harbor the most antibiotic tolerant subpopulations of biofilm cells^48,49^. Thus, aggregates may represent the most protective structures present in a surface-associated community. Therefore, the ability of biofilm communities to produce aggregates may be critical for obtaining the maximal fitness benefits of this growth state.

When CdrA was first described, its function within the biofilm was to maintain the structural integrity of aggregates, in part, by promoting Psl localization to the aggregates periphery^3^. In this study, we show a very similar role for LecB. Both CdrA and LecB are tethered to the outer membrane (CdrA through its outer membrane pore CdrB and LecB is thought to occur at OprF) and bind Psl^3,8,13,50,51^. Otherwise, they are quite distinct from one another, with LecB being a much smaller protein with a clearly defined EPS binding site (no clear EPS binding domain is present in CdrA). In addition, we know that a secreted, extracellular form of CdrA is capable of binding to Psl in the matrix and promoting matrix stability. The simplest interpretation may be that having functionally redundant (or partially redundant) matrix proteins ensures that deleterious mutations targeting either of their genes do not impair the production of aggregates. Redundancy for critical functions is certainly a common theme encountered in *P. aeruginosa* and could partially explain our results.

However, our observations in NB medium indicate that it might not be that simple. In a Δ*lecB* Δ*cdrA* double mutant strain, bacteria remain as a monolayer of cells that largely fail to retain Psl. Complementation with *lecB* restores wild-type biofilm formation, with large aggregates and Psl retained at the aggregate periphery of these aggregates. Curiously, complementation of the double mutant strain with *cdrA* failed to restore production of wild-type aggregates. Psl was retained in the biofilm, but the biofilm was a thick homogenous mat of cells (Fig. 6i, j). This result suggests that under some instances, simple expression of LecB or CdrA is not sufficient to support aggregate production. Why is it unclear? Perhaps, the two proteins differ in their stability under changing environmental conditions. Finally, we cannot rule out that yet unidentified matrix proteins can also contribute to matrix stability in the absence of either LecB or CdrA.

Another point of interest is that two clades of LecB have been proposed, one that groups with PAO1 and another that groups with PA14^23^. Although PA14 cannot synthesize Psl, other members of the PA14 clade can. Whether LecB can serve a similar role in Psl-binding and biofilm structure for other Psl-producing members of the PA14 clade remains to be determined.

We propose the following model to explain our experimental observations. Surface attachment is similar for both strains, resulting in the production of matrix components (EPS and CdrA). At this stage, cells proliferate and produce both CdrA (at low levels) and LecB which leads to the retention of Psl at the base of the biofilm. In the wild-type strain, aggregates continue to grow and biofilm biomass begins to extend beyond the boundary layer and into the overlying bulk fluid. At this stage, *lecB* mutant strains are swept away by the shear stress resulting from fluid flow due to their inability to stabilize the aggregate through Psl interactions. While we are able to determine the consequences of the lack of LecB for biofilm maturation, our data do not explain why LecB and Psl begin to co-localize at the aggregates periphery. Is LecB and/or Psl only produced at the periphery of the aggregates? Does CdrA affect LecB–Psl interactions and vice versa? Our data provide a foundation for the current model, however, there are many questions still left unanswered. Future experimentation will include determining whether *lecB* and Psl expression are coordinately regulated. However, our current knowledge suggests that Psl is primarily influenced by c-di-GMP signaling, while *lecB* is quorum sensing controlled.

In conclusion, our study demonstrates that LecB can serve as a key structural protein in the biofilm matrix. We also demonstrated that Psl and LecB are binding partners and that this interaction impacts biofilm structure. Our findings also have implications for multi-species systems. *P. aeruginosa* may use LecB to adhere to mannose/fucose containing EPS or glycosylated proteins present in established biofilms of other species. We also predict that the converse may be true: *P. aeruginosa* biofilms containing lectins may retain planktonic bacteria of other species that are producing target EPS or capsule. Finally, we predict that lectin production within biofilms may allow *P. aeruginosa* to incorporate free host EPS/oligosaccharides during disease, which might serve as a way of camouflaging the biofilm aggregates from the immune system.

## Methods

### Bacterial strains, media, and growth conditions

Planktonic cultures were routinely grown on Lysogeny broth (LB) medium at 37 °C with constant shaking (225 rpm) unless indicated otherwise. *P. aeruginosa* PAO1 strains and *Escherichia coli* strains used for mutant construction were cultured in LB broth (Fisher Scientific) at 37 °C. For selection and maintenance of plasmids and its derivatives, gentamicin was used at 10 µg/mL for *E. coli* and 30–100 µg/mL for *P. aeruginosa*. Growth curves were performed for all the backgrounds used and no growth defect was observed. All strains are listed in Supplementary Table 1.

### Plasmids, primers, and genetic techniques

Generation of mutants followed the previously published protocol^52^. Briefly, *lecB* flanking regions were produced by PCR amplification from PAO1 genomic DNA using set of primers lecB mut UpF (GATCGAGCTCGGCGACCAGGTGACGCAGTATA)/UpR (CACTCCTTGTGTTGCCATGGTG) and DownF (AACACAAGGAGTGATCAACTGGCCGCTCGGCTA)/DownR(GATCTCTAGACTCGGCTGGTTCTGCCTGTT). These fragments were connected by SOE PCR. *lecB* fragment was inserted into pDONRPEX18Gm using SacI/XbaI sites to produce the deletion construct. Constructs were then conjugated into PAO1 or PAO1 Δ*cdrA*. All mutants were confirmed by sequencing using the primers UpF and DownR. For the creation of the overexpression vector, *lecB* was amplified using the primers lecB over Fw (ATCTGCAGCAGTGGAGATACACCATGGCA) and lecB over Rv (GCACTAGTGAACTCCTAGCCGAGCGG). PCR fragment was then cloned into pJN105 using PstI/SpeI sites. Insert was sequenced using the primer pBAD prom Fw.

### LecB purification

To purify LecB, *P. aeruginosa* harboring pJNLecB was grown overnight in LB containing gentamycin and arabinose. After growth, cells were centrifuged at 10,000 × *g* for 10 min and ressuspended in 100 mM Tris–HCl, 1 mM CaCl_2_, 100 mM NaCl, and pH 8. Bacterial cells were lysed by sonication and centrifuged at 7000 × *g* for 10 min at 4 °C. The supernatant obtained after centrifugation was loaded onto a mannose agarose column (Vector Laboratories). The column was washed with 10 volumes of 100 mM Tris–HCl, 1 mM CaCl_2_, 100 mM NaCl, and pH 8 to remove unspecifically bound proteins. LecB was then eluted with two volumes of 100 mM Tris–HCl, 1 mM CaCl_2_, 500 mM mannose, pH 8. The sample was concentrated by ultrafiltration using Vivaspin (Sartorius, 5 kDa) at 4 °C and dialyzed against 1000 volumes of 100 mM Tris–HCl, 1 mM CaCl_2_, and pH 8 at 4 °C for three times.

### Exopolysaccharide isolation

EPS from PAO1 Δ*wspF*, PAO1 Δ*wspF* Δ*pel*, and PAO1 Δ*wspF* Δ*psl* were precipitated with three volumes of ethanol from the supernatant of overnight cultures. The precipitate was washed with ethanol, air-dried, resuspended in water, treated with DNaseI, RNase A, and proteinase K and subsequently lyophilized. For the purification of Psl these additional steps were followed: first crude preparations were resuspended in water and exhaustively dialyzed using a 3500 MWCO Slide-A-Lyzer dialysis cassette. Then these polysaccharides were fractionated on a Sephadex G-50 column and carbohydrate-containing fractions were screened by western immunoblotting.

### Enzyme-linked immunosorbent assay and lectin assays

Procedures performed here were adapted from previously published protocol^53^. Polysaccharides were coated onto the wells of ELISA plates (Nunc; Maxisorb) by incubation in 250 mM NaCl overnight at 37 °C (50 µl/well). Plates were sealed and incubated in a damp box. Bacterial total sugar extracts were coated at 40 mg/mL. Commercially available bacterial alginate (V-Labs), mucin (Sigma-Aldrich), mannan (Sigma-Aldrich), and purified Psl were coated at 5 mg/mL. After coating, plates were washed five times. All washings and incubations were carried out with 10 mM Tris–HCl, pH 7.5, 10 mM CaCl_2_, 250 mM NaCl, and 0.05% (w/v) Tween 20. The coating efficiency of the different crude extracts was confirmed by titration with anti-Psl-specific antisera. Anti-Psl antibodies were incubated in the wells of coated plates for 1 h at room temperature. Plates were washed five times. Binding was detected by incubation with anti-human IgG Fc-specific, horseradish peroxidase conjugate. Plates were washed five times and developed with Supersignal West Pico Chemiluminescent Substrate (Thermo Scientific). Absorbance was measured using a FluorchemQ. Readings were measured against a blank of uncoated wells. All assays were carried out in triplicate. For lectin-polysaccharide interactions, a modification of the above mentioned technique was used (FLLA). After coating with crude or purified polysaccharides, plates were washed five times. Binding to coated substrate was assessed by incubating 50 uL of FITC-conjugated lectins at 100 µg/mL for 1 h at 37 °C. Plates were washed five times and fluorophore was excited with 485 nm and emission collected at 515 nm.

### Co-immunoprecipitation of LecB and Psl

To assay for LecB binding to Psl, Protein G Dynabeads (Life Technologies, Carlsbad, CA) were incubated with 100 µg/mL anti-LecB polyclonal antibodies (Genscript, Piscataway, NJ) following the manufacturer’s instructions, and then incubated with a mixture of purified Psl and LecB or with only purified LecB (Sigma-Aldrich, St. Louis, MO) for 1 h at 37 °C on a low-speed rotator. Beads were washed three times with TBS plus 0.05% Tween and 1 mM CaCl_2_, and resuspended in Laemmli buffer (Bio-Rad Laboratories, Hercules, CA). Psl was detected in these samples by performing Psl immunoblot as described previously^4^.

We also performed the Co-IP assay with Protein G Dynabeads conjugated with 100 µg/mL of anti-Psl monoclonal antibodies (MEDIMMUNE). Reaction followed very similar steps as mentioned above, where conjugated beads were either incubated with a mixture of purified LecB and Psl or with only purified Psl. After the washes steps and elution of bound material blotting was performed using anti-LecB polyclonal antibodies.

### Anti-LecB and anti-CdrA western blots

anti-LecB and anti-CdrA western blots were performed using antibodies raised against purified LecB (Thermo Fisher) or a synthetic CdrA peptide^8^ (Genscript). Briefly, bacterial cells were resuspended in 100 mM Tris–HCl, 100 mM NaCl, 0.1% Triton X-100, and 0.1% SDS, lysed by sonication, and centrifuged at 7000 × *g* for 10 min at 4 °C. Resulting supernatant was boiled and assayed for protein concentration by Bradford assay (Bio-Rad) for normalization before being loaded onto a 4–20% TGX polyacrylamide gel. Following electroblotting onto a 0.22 µm nitrocellulose membrane, proteins were detected using anti-LecB or anti-CdrA antibodies at 1:10,000 and 1:1000 dilution, respectively. After washes 1:20,000 diluted goat anti-rabbit HRP-conjugated secondary antibodies (Thermo Fisher, Catalog # 65-6120) were used with detection with Supersignal West Pico Chemiluminescent Substrate.

### Isothermal titration calorimetry

ITC experiments were performed with a Microcal ITC-200. The experiments were carried out at 298.15 K. LecB and saccharides were dissolved in 100 mM Tris–HCl pH 7.5 and 1 mM CaCl_2_. LecB was used at 200 μM. A total of 20 injections of 2 μl of saccharide solutions at 2 mM were added at intervals of 200 s while stirring at 500 rev./min. Control experiments performed by injection of saccharides into the buffer solution yielded insignificant heats of dilution, which were used to subtract from the experimental runs. The experimental data were fitted to a theoretical titration curve using software supplied by Microcal. Calculations were performed using the standard equation:

$\Delta G = - RT\;{\mathrm{ln}}\;K = \Delta H - T\Delta S$ where Δ*G*, Δ*H*, and *S* are the changes in free energy, enthalpy, and entropy, respectively, of binding, *T* is the absolute temperature, *R* = 8.32 J · mol^−1^ · K^−1^ and *K* is the association constant. All experiments were performed with *c* values 100 *<* *c* *<* 200 (*c* *=* *K*_a_*M*, where *M* is the initial concentration of the macromolecule).

### Predicted binding of Psl by molecular docking

The X-ray crystal structure of LecB (PAII-L) in complex with D-mannose, PDB ID 1OUR^25^ was used as protein template. The receptor molecule, used in the following local docking, consisted in 23 protein residues within 8 Å of the D-mannose molecule lining the binding site (Ala20-Gln26, Gln43-Asn46; Val69; Ser94-Ala104), 23 waters and two Ca ions. A theoretical model of the pentasaccharide repeat unit of PSL (PSL5) based on NMR data^4,26^ was used as ligand template, linucsID 16756^54,55^.

The *pair_fit* tool, available in PyMol, was used to superimpose the D-mannose unit present in the PSL5 model onto the D-mannose ligand in the crystal structure PDB ID 1OUR. A visual inspection of the resulting LecB-PSL5 local docked model prompted seven of the 23 water molecules to clash or to be too close to PSL5 oligosaccharide, and therefore were manually removed. The LecB-PSL5 model was then submitted to flexible refinement, including optimization of side-chain conformations and rigid-body orientation, using the FireDock web server^56^.

### Flow cell biofilms and confocal microscopy

PAO1-based strains were grown in LB or NB until Log phase and then diluted in NB 1.4% (v/v) or LB 1% to a final OD_600_ of 0.01. PA14 and PAO1 Δ*psl* were grown in LB to mid-log phase and then diluted in modified Jensen’s to a final OD_600_ of 0.05. Flow cell chambers were inoculated with the diluted cultures and incubated inverted for 1 h before initiation of flow. Biofilms, which were continuously supplied with fresh NB 1.4% or modified Jensen’s at 10 mL/h, were grown for 4 days at room temperature. When needed the media was supplemented with 0.05% arabinose. A Zeiss LSM 510 confocal laser scanning microscope (CLSM) was used to image the biofilms and Volocity software (Improvision) was used for image processing. The biomass was stained with 2.5 µM Syto 62 (Molecular Probes) to visualize the entire biomass before imaging. Fluorescent lectins were used to stain the different polysaccharides and were allowed to interact with the biofilm for 15 min. WFL lectin (100 μg/mL; Vector Laboratories) was used for Pel visualization, and HHA lectin (100 μg/mL; EY Laboratories) for Psl. LecB-FITC (Elicityl, Crolles, France) staining followed the same procedure used with HHA and WFL. To determine Psl localization, each condition had nine images from three different experiments analyzed using lines profile from Volocity software (Improvision)^57^.

### Tube biofilms quantification and imaging

Tube biofilms were grown as previously described^58^. Briefly, silicone tubes were inoculated with a suspension of using an OD_600nm_ of 0.1 and grown at room temperature using NB 1.4% with or without 0.2% arabinose at flow rate of 10 mL/h for 6 days. At the end of the experiment, flow was interrupted, inlet was blocked and outlet was disconnected. Tubes were placed vertically inside a conical tube and inlet was quickly unblocked, draining the contents inside the tube by gravity. This fraction was called non-adherent. An aliquot was used to determine colony forming units, while the remaining sample was centrifuged and filtered to separate cells from EPS. Supernatant containing only released Psl was used for the immunoblot with anti-Psl antibodies.

The material still adhered to the tube walls was physically removed using an L-shaped loop and this material was resuspended in 500 µL of sterile 1× PBS. This fraction was named adherent and we applied the same treatment described above.

To image the tube biofilms by confocal microscopy we performed the same steps until draining the tube. After that, we cut the first centimeter of tubing and used the subsequent 2.4 cm of tube for confocal microscopy. The tube was opened longitudinally and carefully placed in the well of a 12-well plate containing 2 mL of sterile media. A Zeiss LSM 800 CLSM was used to image the biofilms and Volocity software (Improvision) was used for image processing. The biomass was stained with 2.5 µM Syto 62 (Molecular Probes) to visualize the entire biomass before imaging.

### Reporting summary

Further information on research design is available in the Nature Research Reporting Summary linked to this article.

## Supplementary information

Supplementary Information

Description of Additional Supplementary Files

Supplementary Movie 1

Supplementary Movie 2

Supplementary Movie 3

Reporting Summary

Source Data

## Data Availability

The authors declare that data supporting the findings of this study are available within the paper, in its supplementary information files and its source file. The source data underlying Figs. 1a–c, 6b,d, f, h and j, 7b and c and Supplementary Figs. 5 and 7 and Table 1 are provided as a Source Data file.
