# Supplementary material for: The Pseudomonas aeruginosa lectin LecB binds to the exopolysaccharide Psl and stabilizes the biofilm matrix
Source: Nat Commun. 2019 May 16;10:2183. doi: 10.1038/s41467-019-10201-4 (PMC6522473; doi:10.1038/s41467-019-10201-4)
Supplement: Supplementary file 1 — Supplementary Information [file 41467_2019_10201_MOESM1_ESM.pdf]

Supplementary Information

The *Pseudomonas aeruginosa* lectin LecB binds to Psl and stabilizes the biofilm matrix

Passos da Silva D. *et al.*

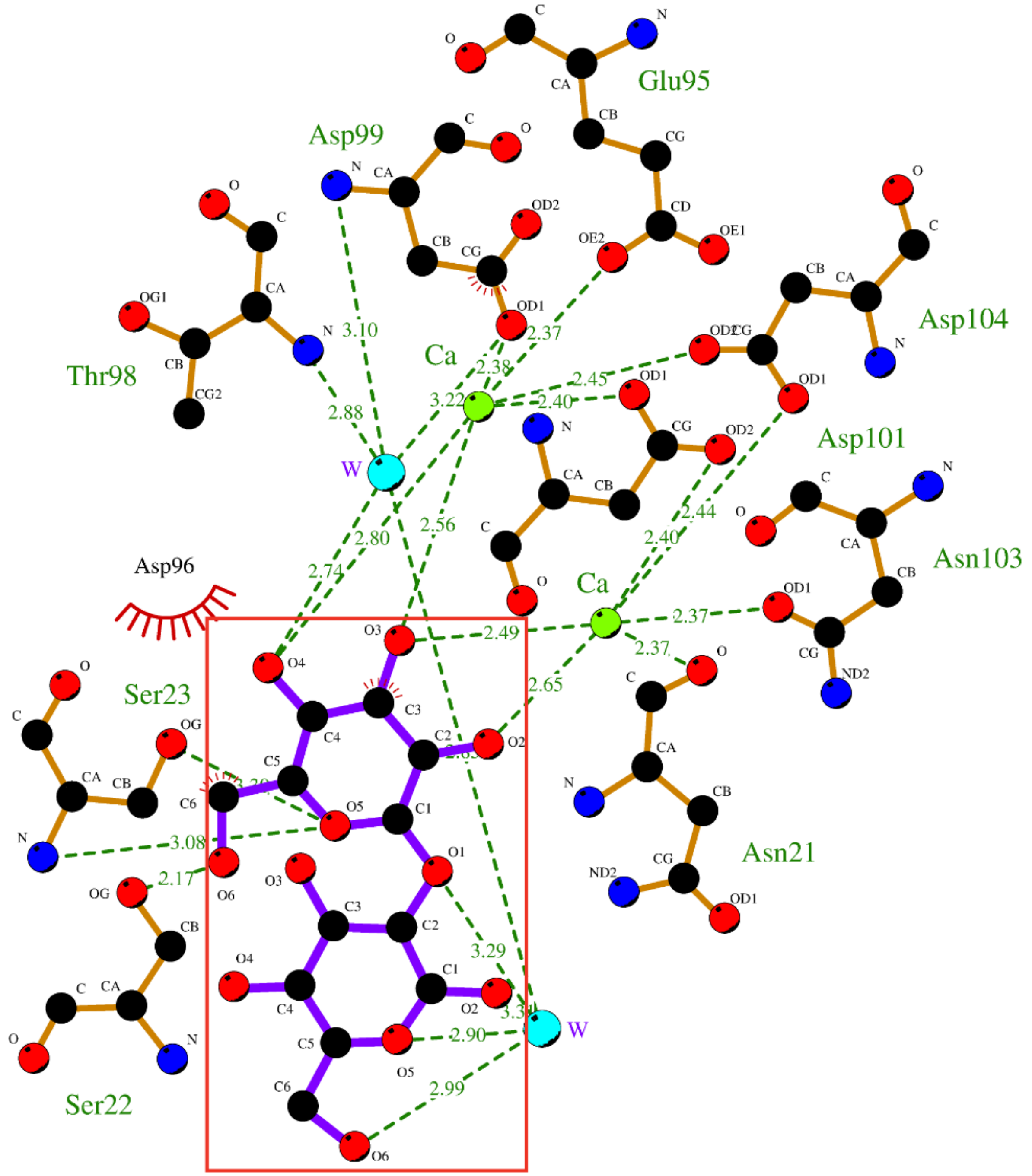

Supplementary Figure 1. The binding mode of LecB interacting with α-1,2' mannobiose. The disaccharide is highlighted in the red box, while water molecules are represented in cyan, Ca^{+2} in green and the remaining are LecB amino acids involved in the interaction.

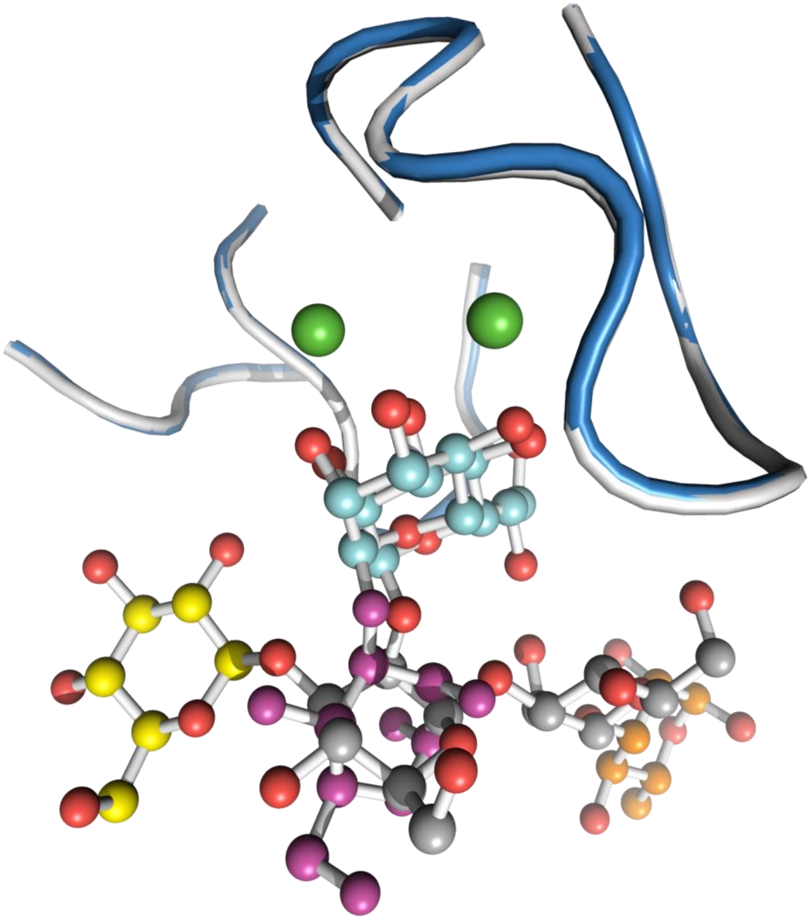

Supplementary Figure 2. Superimposition of PAO1 LecB-Psl and PA14 LecB- α-1,3' mannobiose interactions. PAO1 LecB is represented in white, while PA14 LecB is represented in blue. For the saccharides, the pentasaccharide from Psl is represented as α-D-mannopyranose in cyan, β-D-mannopyranose in grey, α-L-rhamnopyranose in orange, and β-D-glucopyranose in yellow, while the disaccharide α-1,3' mannobiose is represented in cyan and violet.

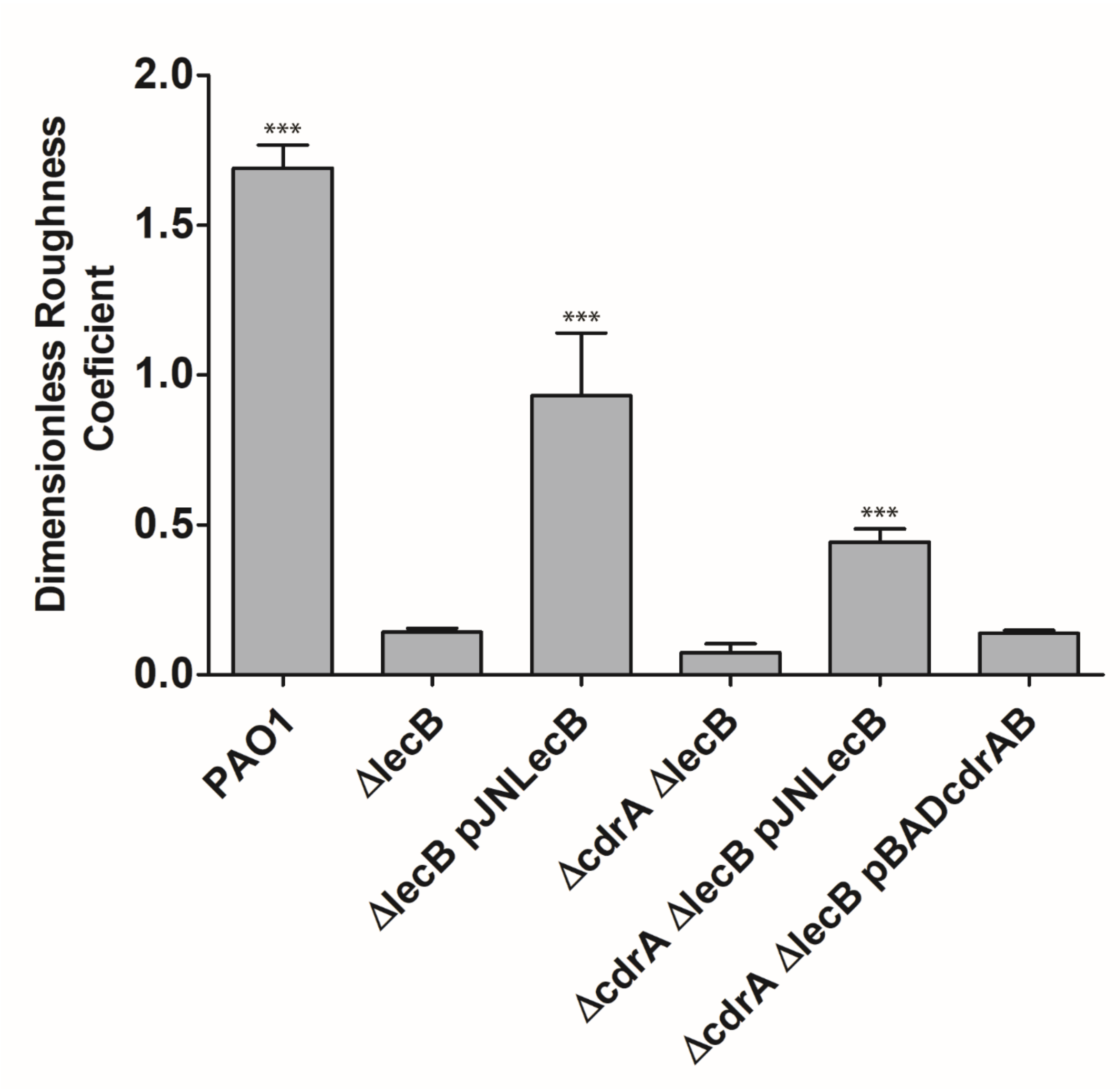

Supplementary Figure 3. LecB presence leads to increased roughness of biofilms. The presence of complex structures in a biofilm can be determined by the roughness measurements from COMSTAT. We observed that in the absence of LecB, (Δ*lecB,* Δ*cdrA* Δ*lecB,* and Δ*cdrA* Δ*lecB* pBADcdrAB) biofilms possessed roughness close to zero, meaning a very smooth surface, the introduction of lecB leads to increased roughness, which can be interpreted with a more uneven surface characterized by the presence of aggregates. All COMSTAT measurements were performed in 9 images from 3 different experiments per condition. *** $p < 0.0001$, *t*-test, n > 3.

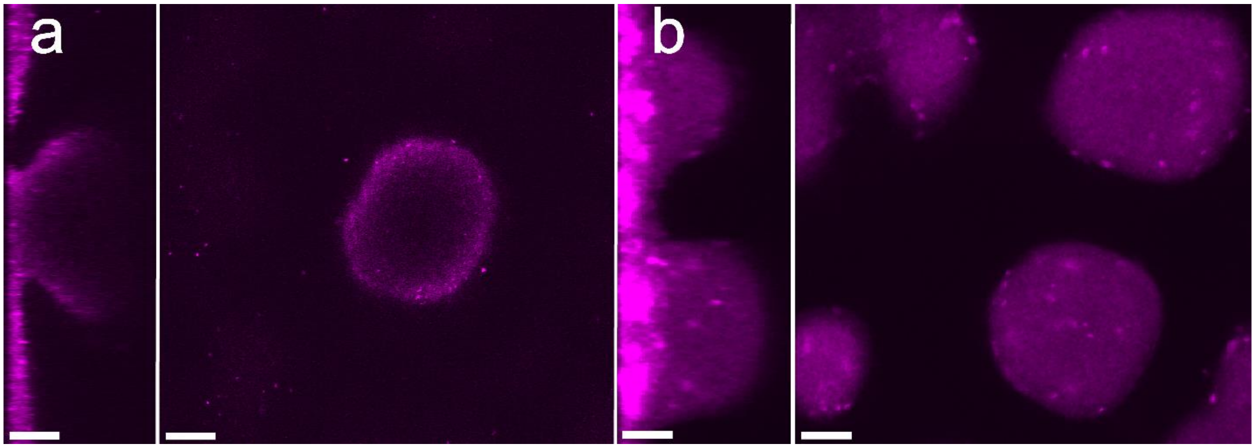

Supplementary Figure 4. PAO1 Δ*lecB* does not display a defective phenotype when grown in LB. (a) 4 day-old biofilm of PAO1 grown in LB 1%. (b) 4 day-old biofilm of PAO1 Δ*lecB* grown in LB 1%. Scale bars = 25 µm.

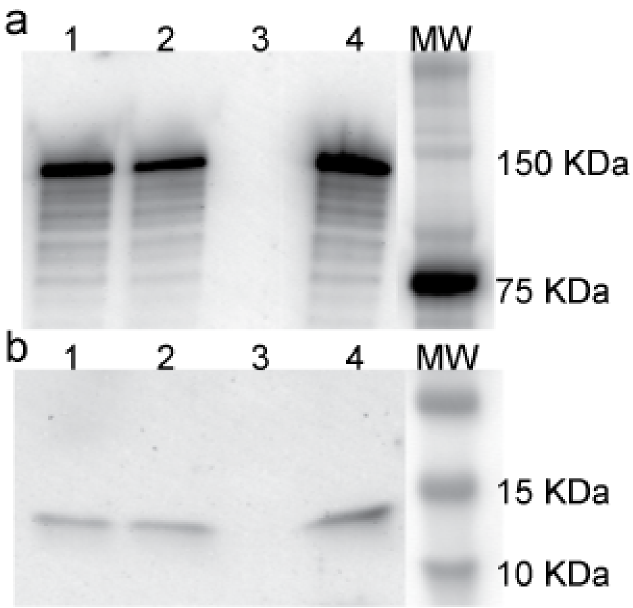

Supplemntary Figure 5. CdrA levels are reduced in biofilms grown in NB. (a) Western blot from 6 day-old tube biofilms developed using anti-CdrA. (1) PAO1 grown in LB, (2) PAO1 grown in NB, (3) PAO1 Δ*cdrA* grown in LB, and (4) purified CdrA. (b) Western blot from 6 day-old tube biofilms developed with anti-LecB. (1) PAO1 grown in LB, (2) PAO1 grown in NB, (3) PAO1 Δ*lecB* grown in LB, and (4) purified LecB.

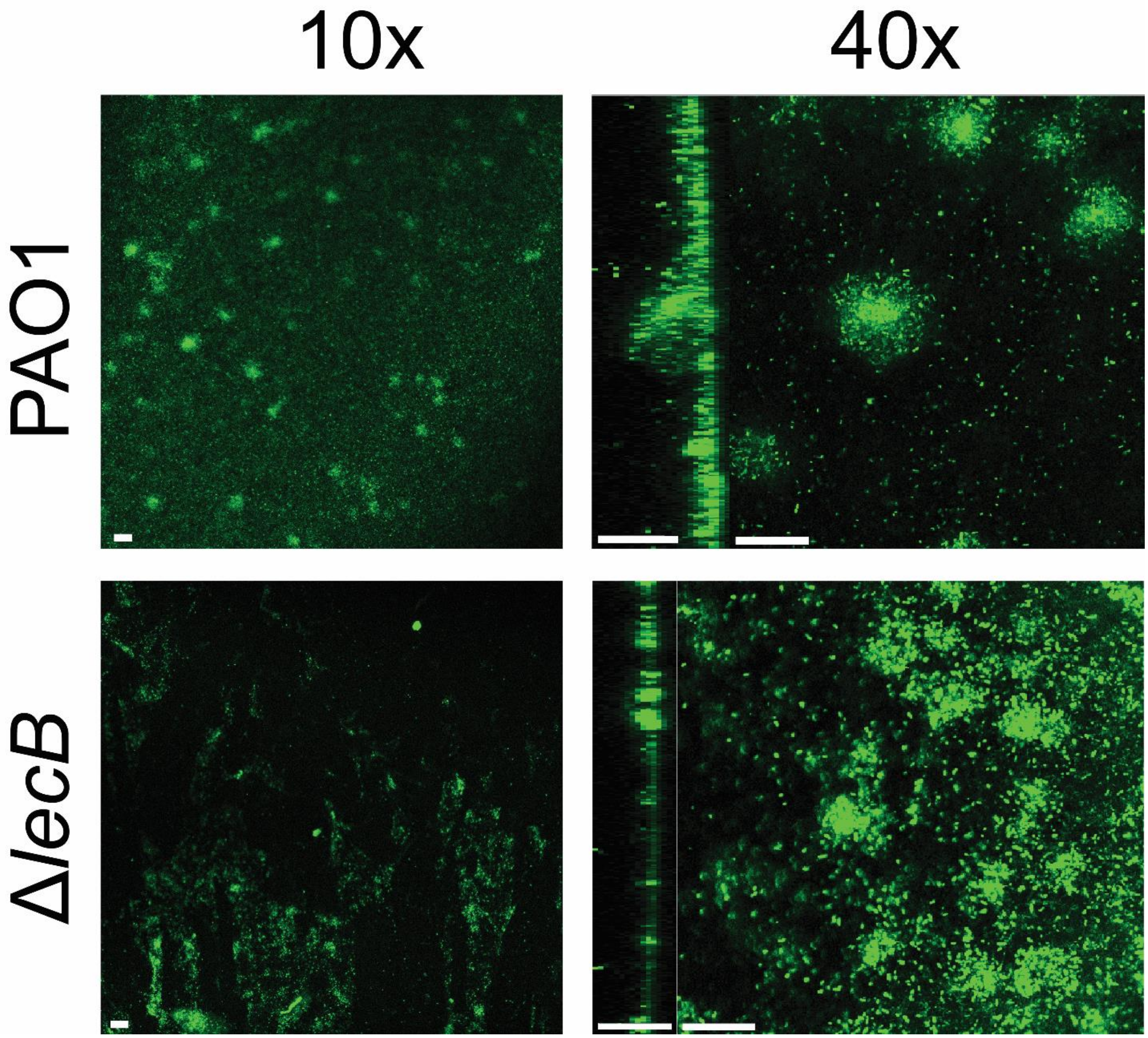

Supplementary Figure 6. LecB mutant display defective mature aggregate formation in silicone tube biofilms. PAO1 6-day old biofilm display the surface completely covered by cells with the presence of mature aggregates, while Δ*lecB* biofilms are characterized by zones void of cells and zones containing microaggregates. Scale bars = 25 µm.

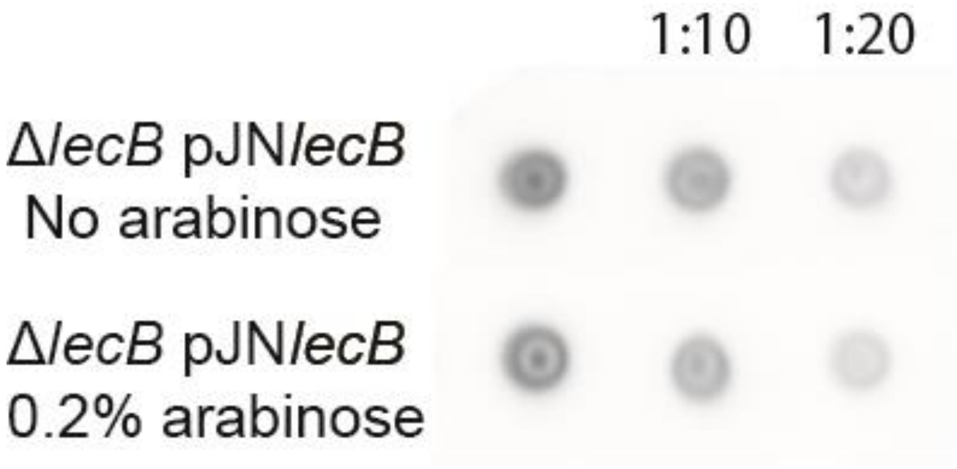

Supplementary Figure 7. Adherent fraction shows the same levels of Psl retention on tube biofilms. Dot blot of adherent fractions derived from Δ*lecB* pJNLecB without or with 0.2% arabinose 6-day old tube biofilms. Immunodetection was performed using anti-Psl antibodies. Expression of *lecB* does not affect the levels of retained Psl when compared to the uninduced condition.

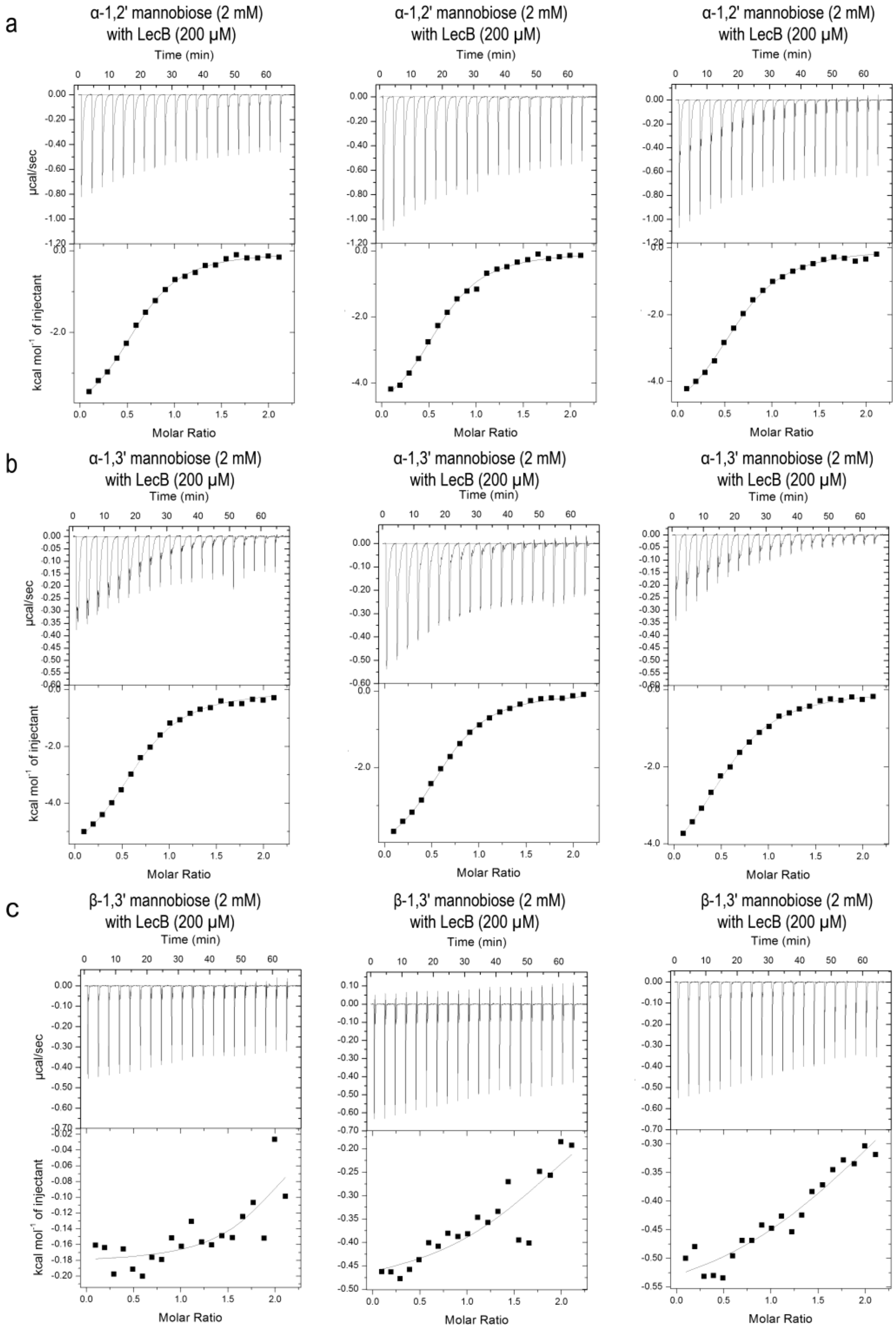

Supplementary Figure 8. LecB possess binding affinty towards α-mannobiose, but not β-mannobiose. ITC titration data of (a) α-1,2' mannobiose (Psl side chain) into purified LecB, (b) α-1,3' mannobiose into purified LecB (not present in Psl) and (c) β-1,3' mannobiose (Psl linear chain) into purified LecB.

Supplementary Table 1. Bacterial strains, plasmids and primers used in this study.

Bacterial strains	Relevant genotype or characteristics	Source
PAO1	Wild type	[1]
PA14	Wild type	[2]
PAO1 Δ*psl*	Δ*pslBCD*; markless	[3]
PAO1 Δ*cdrA*	Δ*cdrA*	[4]
PAO1 Δ*lecB*	Δ*lecB*; markless	This study
PAO1 Δ*cdrA* Δ*lecB*	Δ*cdrA* Δ*lecB*; markless	This study
**Plasmids**		
pJN105	araC-PBAD cassete cloned into pBBR1MCS5, Gm^r	[5]
pJNLecB	*lecB* cloned into pJN105, Gm^r	This study
pMJT-1	araC-PBAD cassete from pJN105 cloned in pUCP18, Ap^r	[6]
pBADCdrAB	*cdrA* and *cdrB* cloned into pMJT-1, Ap^r	[4]
**Primers**	**Sequence**	
lecB UpF	GATCGAGCTCGGCGACCAGGTGACGCAGTATA	
lecB UpR	CACTCCTTGTGTTGCCATGGTG	
lecB DownF	AACACAAGGAGTGATCAACTGGCCGCTCGGCTA	
lecB DownR	GATCTCTAGACTCGGCTGGTTCTGCCTGTT	
lecB over Fw	ATCTGCAGCAGTGGAGATACACCATGGCA	
lecB over Rv	GCACTAGTGAACTCCTAGCCGAGCGG	

Supplementary References

1 Holloway, B. W. Genetic recombination in Pseudomonas aeruginosa. *Journal of general microbiology* **13**, 572-581, doi:10.1099/00221287-13-3-572 (1955).

2 Rahme, L. G. *et al.* Common virulence factors for bacterial pathogenicity in plants and animals. *Science* **268**, 1899-1902 (1995).

3 Kirisits, M. J., Prost, L., Starkey, M. & Parsek, M. R. Characterization of colony morphology variants isolated from Pseudomonas aeruginosa biofilms. *Applied and environmental microbiology* **71**, 4809-4821, doi:10.1128/AEM.71.8.4809-4821.2005 (2005).

4 Borlee, B. R. *et al.* Pseudomonas aeruginosa uses a cyclic-di-GMP-regulated adhesin to reinforce the biofilm extracellular matrix. *Mol Microbiol* **75**, 827-842, doi:10.1111/j.1365-2958.2009.06991.x (2010).

5 Newman, J. R. & Fuqua, C. Broad-host-range expression vectors that carry the L-arabinose-inducible Escherichia coli araBAD promoter and the araC regulator. *Gene* **227**, 197-203 (1999).

6 Kaneko, Y., Thoendel, M., Olakanmi, O., Britigan, B. E. & Singh, P. K. The transition metal gallium disrupts Pseudomonas aeruginosa iron metabolism and has antimicrobial and antibiofilm activity. *The Journal of clinical investigation* **117**, 877-888, doi:10.1172/JCI30783 (2007).
