# Supplementary material for: The Pseudomonas aeruginosa lectin LecB binds to the exopolysaccharide Psl and stabilizes the biofilm matrix
Source: Nat Commun. 2019 May 16;10:2183. doi: 10.1038/s41467-019-10201-4 (PMC6522473; doi:10.1038/s41467-019-10201-4)
Supplement: Supplementary file 2 — Description of Additional Supplementary Files [file 41467_2019_10201_MOESM2_ESM.pdf]

**Description of Additional Supplementary Files**

File Name: Supplementary Movie 1
Description: Aggregates formed by PAO1 are resistant to flow perturbations. A movie of PAO1 flow cell-grown biofilms (96 h in NB 1.4%) subjected to continuous flow during the course of the movie (10 mL/min)

File Name: Supplementary Movie 2
Description: Underdeveloped aggregates formed by ΔlecB partially are susceptible to flow perturbations. A movie of PAO1 ΔlecB flow cell-grown biofilms (96 h in NB 1.4%) subjected to continuous flow during the course of the movie (10 mL/min)

File Name: Supplementary Movie 3
Description: Aggregates formed by ΔcdrA ΔlecB are easily displaced by shear stress. Effect of flow (10 mL/min) on the structure of ΔcdrAΔlecB biofilms grown for 96h in NB 1.4%

.
